# Spatial distribution and core community of diazotrophs in Biological soil crusts and subsoils in temperate semi-arid and arid deserts of China

**DOI:** 10.3389/fmicb.2023.1074855

**Published:** 2023-08-07

**Authors:** Kai Tang, Yungang Liang, Bo Yuan, Jianyu Meng, Fuying Feng

**Affiliations:** ^1^Laboratory for Environmental Microbiology and Biotechnology in Arid and Cold Regions, College of Life Sciences, Inner Mongolia Agricultural University, Hohhot, China; ^2^College of Life Science, Inner Mongolia Normal University, Hohhot, China

**Keywords:** *Skermanella*, *Nostoc* and *Scytonema*, core diazotroph community, available nitrogen content, Biological soil crusts, temperate semi-arid and arid deserts

## Abstract

**Introduction:**

Biological soil crusts (BSCs) are distributed in arid and semiarid regions, and they function as important microhabitats for nitrogen fixation. The diazotroph community is critical for nitrogen fixation in BSCs and their subsoils. However, little is known about the key groups in different types of BSCs and subsoils in temperate semi-arid or arid deserts.

**Methods:**

Here, we sampled three types of BSCs and their subsoils from the Inner Mongolian plateau, investigated the distribution characteristics of the diazotroph community by high-throughput sequencing, predicted keystone species using the molecular ecological network analyses pipeline (MENAP), and verified their close relationship with the available nitrogen (AN) content.

**Results:**

The results showed that available nitrogen content in BSCs was higher than that in subsoils in three different types of BSCs, and there were differences among seasons and according to the mean annual precipitation. The abundance of diazotrophs was higher in Cyano-BSCs, while diversity had no significant difference among BSCs and subsoils. Cyanobacteria and Proteobacteria, Nostocaceae and Scytonemataceae, *Skermanella*, *Scytonema*, *Azohydromonas*, *Nostoc* and *Trichormus* were the dominant phyla, families, and genera, respectively. The dominant groups belong to Skermanella, Scytonema, and Nostoc formed the core diazotroph community in the three types of BSCs and subsoils, and each had a close relationship with AN.

**Discussion:**

These results indicate that diazotrophs in BSCs and subsoils had high diversity, and the core diazotroph communities have a close relationship with nitrogen fixation and that they may be the main contributor to nitrogen fixing in BSCs and subsoils in temperate deserts.

## 1. Introduction

Nitrogen (N) is an essential element for all living organisms, as it is involved in the biosynthesis of key cellular components (Kuypers et al., 2018); it is generally the second largest major factor (next to water) limiting primary production in desert ecosystems (Vitousek and Howarth, 1991; Gebauer and Ehleringer, 2000; Ramond et al., 2022). Knowledge of the factors controlling N input is critical for understanding nitrogen cycles in dryland ecosystems.

Biological soil crusts (BSCs) are specialized microbial communities binding with soil particles that form at the soil surface in drylands, which cover approximately 40% of the global terrestrial surface (Belnap, 2003; Rodriguez-Caballero et al., 2022). They are prominent in arid lands, protecting soils against erosion and creating “islands of fertility” (Belnap et al., 2016). Nitrogen fixation is an important source of nitrogen for supporting desert primary productivity (Evans and Belnap, 1999; Belnap et al., 2001). As major nitrogen fixation microhabitat, BSCs may supply nearly half of the fixed N in natural ecosystems (Elbert et al., 2012; Barger et al., 2016).

Presently, most of studies about diazotrophs in BSCs focused on the topsoil. Diazotrophs in the domains bacteria and archaea are known to be capable of fixing nitrogen, and they include free-living diazotrophs as well as those associated with lichen and bryophytes in symbiosis with lichen and moss crusts (Rikkinen et al., 2002; Adams and Duggan, 2008). Motile non-heterocystous cyanobacteria (for example, *Microcoleus vaginatus* or *M. steenstrupii*) are numerically dominant in BSCs diazotroph communities; heterocystous cyanobacteria (for example, *Scytonema*, *Spirirestis* and *Nostoc*) increase in abundance during crust succession and are abundant in mature crusts (Yeager et al., 2007, 2012). Heterocystous cyanobacterial genera (e.g., *Anabaena*, *Calothrix*, *Cylindrospermum*, *Dicothrix*, *Hapalosiphon*, *Nodularia*, and *Nostoc*), non-heterocystous genera (e.g., *Lyngbya*, *Microcoleus*, *Oscillatoria*, *Phoridium*, and *Tolypothrix*), could efficiently elevate soil N availability through nitrogen fixation in desert ecosystems (Belnap, 2002; Thomazo et al., 2018). Members of Clostridiaceae and Proteobacteria, some non-cyanobacterial diazotrophs, may also be important dinitrogen fixers in BSCs during early BSC formation (Pepe-Ranney et al., 2015). The features of soil under BSCs are distinctly different from BSCs, with lower light intensity, oxygen concentration and soil nutrient contents (Belnap, 2003; Belnap et al., 2016), which means diazotrophs should differ from the corresponding BSCs. However, diazotroph communities in subsoils beneath different types of BSCs remain to be characterized.

The core microbiome, also regularly named by keystone taxa, are the taxa which have major influence on microbiome composition and function at a particular space or time (Van De Water et al., 2016; Lemanceau et al., 2017; Banerjee et al., 2018). They comprised microorganisms commonly present in hosts or within particular niches of a broad host community (Turnbaugh et al., 2007). Previous studies that have defined core plant microbiomes by abundance, occupancy, and both (Shade and Stopnisek, 2019). In macroecology, abundance-occupancy distributions are commonly used to think about changes in diversity over space (Shade et al., 2018; Grady et al., 2019; Zhang et al., 2022). This principle was employed to determine the core diazotroph community in BSCs and subsoils in this study.

Therefore, we hypothesized that: (a) the diazotroph communities were different in abundance, diversity and structure among three types of BSCs and their subsoils, (b) core diazotroph communities were the key in accumulation of available nitrogen during BSCs succession. To test these hypotheses, three types of BSCs (Cyano-, Lichen-, and Moss-BSCs) and their subsoils were collected from deserts in the western region of the Inner Mongolia plateau, China. We analyzed the diazotroph community structure based on the *nif*H gene by high-throughput sequencing technology. We then evaluated the differences in diazotroph community features and core diazotroph communities during BSCs succession and analyzed the correlations between the core diazotroph community and soil available nutrient contents.

## 2. Materials and methods

### 2.1. Study site description

**Figure 1:**
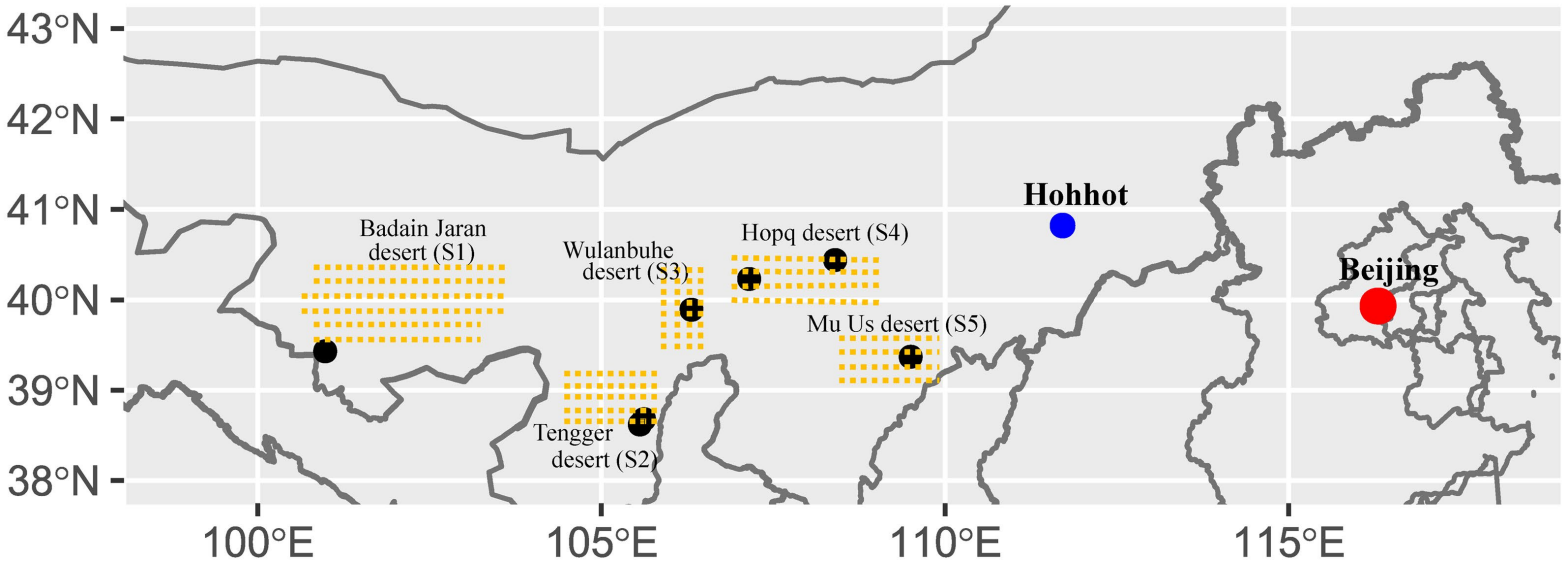
Location of sampling sites in different deserts. S1, Badain Jaran desert; S2, Tengger desert; S3, Wulanbuhe desert; S4, Hopq desert; S5, Mu Us desert. There are two sampling sites located in the Tengger desert.

The Inner Mongolia plateau is the second largest plateau in China, and BSCs are widely distributed in the major western deserts. Cyano-BSCs, Lichen-BSCs, and Moss-BSCs are found in the region, and our seven sampling sites were located in five deserts (Figure 1). The natural landscape consists of fixed and semi-fixed dunes covered by low desert vegetation and patches of BSCs. According to records from the nearby weather station, the annual mean temperature ranges from 6.67 to 10.10°C; the annual mean precipitation ranges from 82 to 338 mm and varies widely among different deserts. All sampling sites had high sunlight time (above 2800 h/year). The meteorological data of sampling sites were obtained from the China Integrated Meteorological Information Sharing System (CIMISS).^1^

### 2.2. Sample collection and available nitrogen analysis

The sampling sites were distributed in five deserts in semi-arid and arid regions of Northwestern China, namely Badain Jaran (S1), Tengger (S2), Wulanbuhe (S3), Hopq (S4) and Mu Us (S5) deserts from the western to eastern (Figure 1 and Supplementary Table S1). Average annual rainfall gradually decreases extending from the eastern to western regions. BSCs are widely distributed in these deserts at various successional stages (Tang et al., 2021). We sampled BSCs and their subsoils (about 2 cm beneath BSCs) with a sterile knife after removing the withered grass and fallen leaves, including eight Cyano-BSCs, nine Lichen-BSCs, eleven Moss-BSCs, and their corresponding subsoils (10 cm × 10 cm per sample, separated by at least 20 m) (Supplementary Table S1). The sample collection was performed in May 2015 and September 2016. The geographic location of each sample was recorded by a Global Positioning System (GPS) recorder (Garmin eTrex Venture, Garmin Ltd., Olathe, Kansas, United States). One portion was used to measure the content of available nitrogen (AN) and was air-dried at room temperature for no more than 3 days, and determined by the alkaline diffusion method (ISSCAS and IoSS, 1978); while the other portion was used to extract DNA and was stored at −70°C for up to 2 weeks. The detailed data of soil physicochemical properties, geographic location parameters, and meteorology were referred from our previous work (Tang et al., 2021).

### 2.3. Quantitative PCR analysis

The molybdenum-iron (MoFe) nitrogenase enzyme ([1.18.6.1]) can fix dinitrogen into ammonia, and as such, it is the key enzyme of nitrogen-fixing microorganisms in the environment (Kuypers et al., 2018). The *nif*H gene is the key gene in coding the nitrogenase enzyme, and this gene was used to quantify the absolute abundance of diazotroph communities. Quantitative PCR (q-PCR) was conducted to evaluate *nif*H gene abundance in diazotroph bacteria in different samples. *nif*H gene fragments were amplified by primers *pol*F (5′-TGCGAYCCSAARGCBGACTC-3′)/*pol*R (5′-ATSGCCATCATYTCRCCGGA-3′) (product size about 300 bp) (Brankatschk et al., 2012). The target DNA fragments were amplified and cloned into a pEASY-T1 vector (TransGen, China), and then, the recombinant plasmid was further confirmed by sanger sequencing method using general primers. Quantification of the recombinant plasmid was performed using a NanoDrop 2000 UV–vis spectrophotometer (Thermo Fisher Scientific, Wilmington, DE, United States). The qPCR standard curve for the *nif*H gene was generated using 10-fold serial dilutions of a plasmid containing the *nif*H gene fragment inserts. Each qPCR reaction contained 10 μL of SYBR Premix Ex Taq^™^ (Takara, Dalian, China), 0.4 μL of 10 mM primers (each), 7.2 μL of sterilized MilliQ water, and 2 μL of standard or extracted soil DNA. The PCR used the following program: initial denaturation at 95°C for 1 min, followed by 40 cycles of 95°C for 15 s/10 s for denaturation, 58°C for 15 s/20 s for annealing and elongation, and a final cycle at 60°C for 30 s for cooling. All analyses were carried out with a thermocycler (Roche Light Cycler 480 real-time PCR). A standard curve for the *nif*H gene (*y* = −0.2926x + 12.169, *R*^2^ = 0.997) was obtained and used to calculate the absolute abundance of *nif*H genes. The melting curve was obtained to confirm that the amplified products were of the appropriate size.

### 2.4. DNA extraction, sequencing, and data processing

Microbial DNA was extracted from samples using the E.Z.N.A.^®^ soil DNA Kit (Omega Bio-tek, Norcross, GA, United States) according to the manufacturer’s protocols. The final DNA concentration and purification were determined using a NanoDrop 2000 UV–vis spectrophotometer (Thermo Fisher Scientific, Wilmington, DE, United States), and DNA quality was checked by 1% agarose gel electrophoresis. The *nif*H genes were amplified with the primers *nif*HF (5′-AAAGGYGGW ATCGGYAARTCCACCAC-3′)/*nif*HR (5′-TTGTTSGCSGCRTACATSGCCA TCAT-3′) (Rösch et al., 2002). The PCR reactions were conducted using the following program: 3 min of denaturation at 95°C, 27/25 cycles of 30 s at 95°C, 30 s for annealing at 55/58°C, 45 s for elongation at 72°C, and a final extension at 72°C for 10 min. PCR reactions were performed in triplicate: 20 μL mixtures containing 4 μL of 5 × Fast Pfu Buffer, 2 μL of 2.5 mM dNTPs, 0.8 μL of each primer (5 μM), 0.4 μL of Fast Pfu polymerase, and 10 ng of template DNA. The resulting PCR products were extracted from 1% agarose gels, further purified using an AxyPrep DNA Gel Extraction Kit (Axygen Biosciences, Union City, CA, United States), and quantified using QuantiFluor^™^-ST (Promega, United States) according to the manufacturer’s protocol. Purified amplicons were pooled in equimolar volumes and paired-end sequenced (2 × 300) on an Illumina MiSeq platform (Illumina, San Diego, United States) according to standard protocols by Majorbio Bio-Pharm Technology Co. Ltd. (Shanghai, China). The raw reads were deposited into the NCBI Sequence Read Archive (SRA) database, with the BioProject accession number of PRJNA649746.

Raw fastq files were demultiplexed, quality filtered by Trimmomatic (v3.02) (Bolger et al., 2014), and merged by FLASH with the following criteria: (i) the reads were truncated at any site receiving an average quality score < 20 over a 50 bp sliding window. (ii) Primers were exactly matched allowing two nucleotide mismatches, and reads containing ambiguous bases were removed. (iii) Sequences whose overlap was longer than 10 bp were merged according to their overlap sequence.

Operational taxonomic units (OTUs) were clustered with a 95% (*nif*H gene) (Carrell et al., 2019) similarity cut-off using UPARSE (version 7.1)^2^ (Edgar, 2013), and chimeric sequences were identified and removed using UCHIME (Edgar et al., 2011). The taxonomy of each *nif*H gene was analyzed by the RDP Classifier algorithm^3^ against the fgr/*nif*H database using a confidence threshold of 70%.

### 2.5. Co-occurrence network construction and analysis

Co-occurrence networks were constructed to predict the core diazotrophs in BSCs and subsoils. The co-occurrence networks of diazotroph bacteria were analyzed using the molecular ecological network analyses pipeline (MENAP) at http://ieg4.rccc.ou.edu/mena/, following the default settings (only the OTUs detected in more than half of the total samples) (Deng et al., 2012). The co-occurrence networks were constructed following a Random Matrix Theory (RMT)-based approach with the default cut-off. The mean average degree (avgK), average path distance (GD), and average clustering coefficient (avgCC) described the overall features of the co-occurrence networks (Deng et al., 2012). Network graphs were visualized by Cytoscape 3.7.2 (Shannon et al., 2003).

### 2.6. Statistical analysis

One-way analysis of variance and Tukey’s test were carried out using SPSS 19.0 software (SPSS Inc., Chicago, IL, United States) to determine the differences in available nitrogen (AN) content, *nif*H copy numbers and the Shannon index was calculated for different types of BSCs and their subsoils. The figures were generated with GraphPad Prism 7.0 (GraphPad Software Inc., San Diego, CA, United States).

Structural equation models (SEMs) were constructed with the Amos 21.0 software (SPSS Inc., IBM Co., Armonk, NY, United States) to evaluate direct and indirect paths through which soil carbon content (SOC) and AN content influenced diazotroph community structures. Model adequacy was evaluated through χ^2^ tests, the comparative fit index (CFI), and root mean square error of approximation (RMSEA). Low χ^2^ values (*p* > 0.05), high CFI (>0.90), and low RMSEA (<0.08) indicated sound fits of the model (Hooper et al., 2008).

Multivariate analysis by linear models analysis (MaAsLin) is a multivariate linear modeling tool with boosting that tests for associations between specific microbial taxa and metadata, which could reduce the total amount of correlations to be tested, and allow for improvements in the robustness of the additive general linear models (Mallick et al., 2021). In this study, MaAsLin was run with defaults parameters to identify association of diazotrophs in different taxonomy level (Genus, species, and operational taxonomic unit) with the content of AN in three types of BSCs and their subsoils.

## 3. Results

### 3.1. Available nitrogen contents in BSCs and subsoils

**Figure 2:**
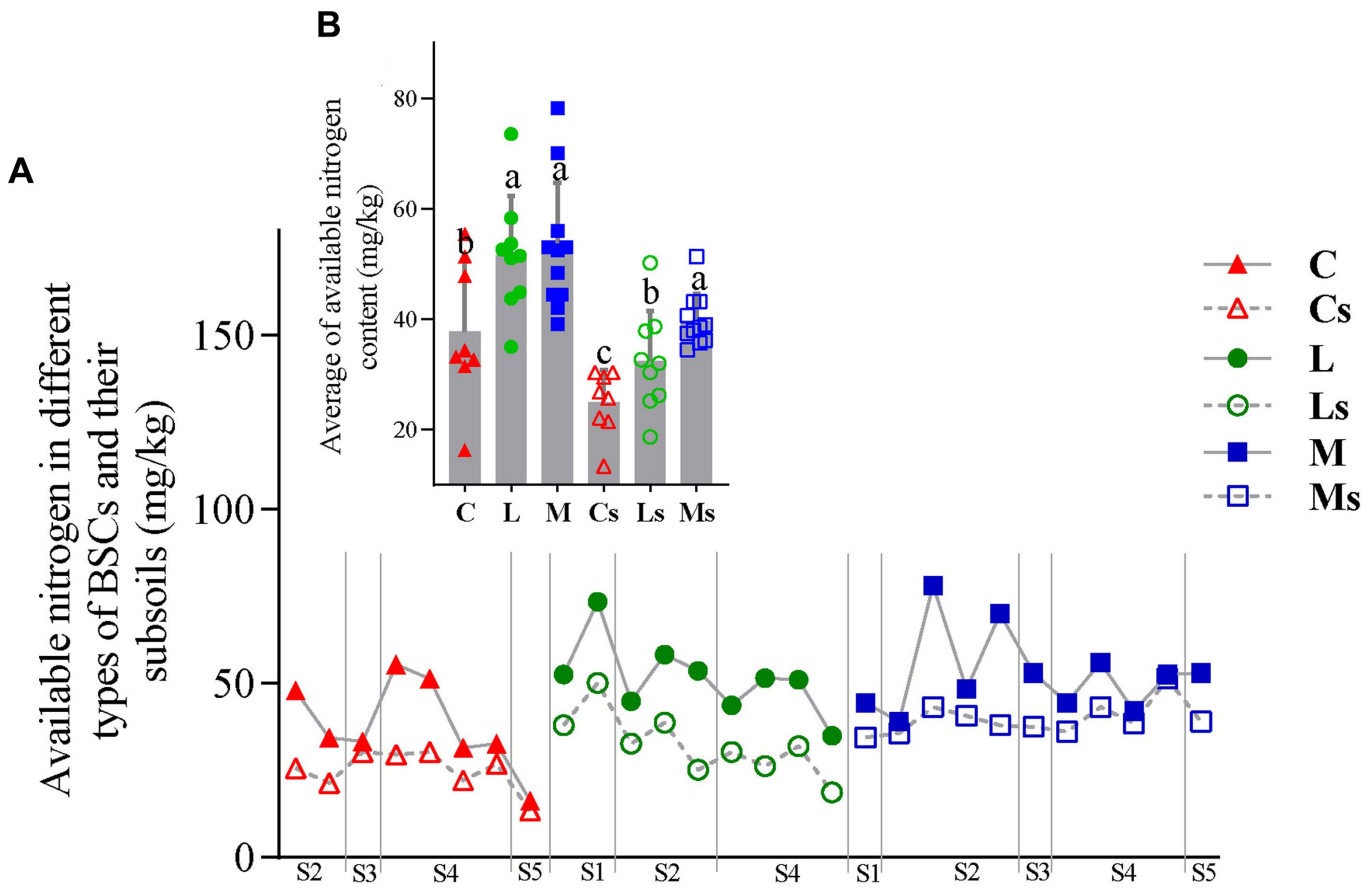
Change in available nitrogen content in different types of Biological soil crusts (BSCs) and their subsoils **(A)**, and their intragroup difference **(B)**. C, L, and M correspond to Cyano-, Lichen-, and Moss-BSCs, respectively. An “s” letter in the sample name indicates subsoil samples. The *nif*H copy numbers and Shannon index differences between groups were evaluated with ANOVA tests (*p* < 0.05). S1, Badain Jaran desert; S2, Tengger desert; S3, Wulanbuhe desert; S4, Hopq desert; S5, Mu Us desert.

The contents of AN in the three types of BSCs were higher than those of their corresponding subsoils (Figure 2A). The contents of BSCs were higher than those of their subsoils. The highest AN content occurred in Moss-BSCs (78.17 mg/kg) located in Tengger desert (Figure 1 and S2 site), then Lichen-BSCs and Cyano-BSCs (Figure 2B and Supplementary Table S1). Similarly, the highest AN content of subsoils was 51.33 mg/kg also observed in Moss-S (Ms) but located in Hopq desert (Figure 1 and S4 site), and the average AN in Moss-S (Ms) was 30.08 and 21.67% significantly higher than those of Cyano-S (Cs) and Lichen-S (Ls) (*p* < 0.05), respectively (Figure 2B).

### 3.2. Absolute abundances of diazotrophs in BSCs and subsoils

**Figure 3:**
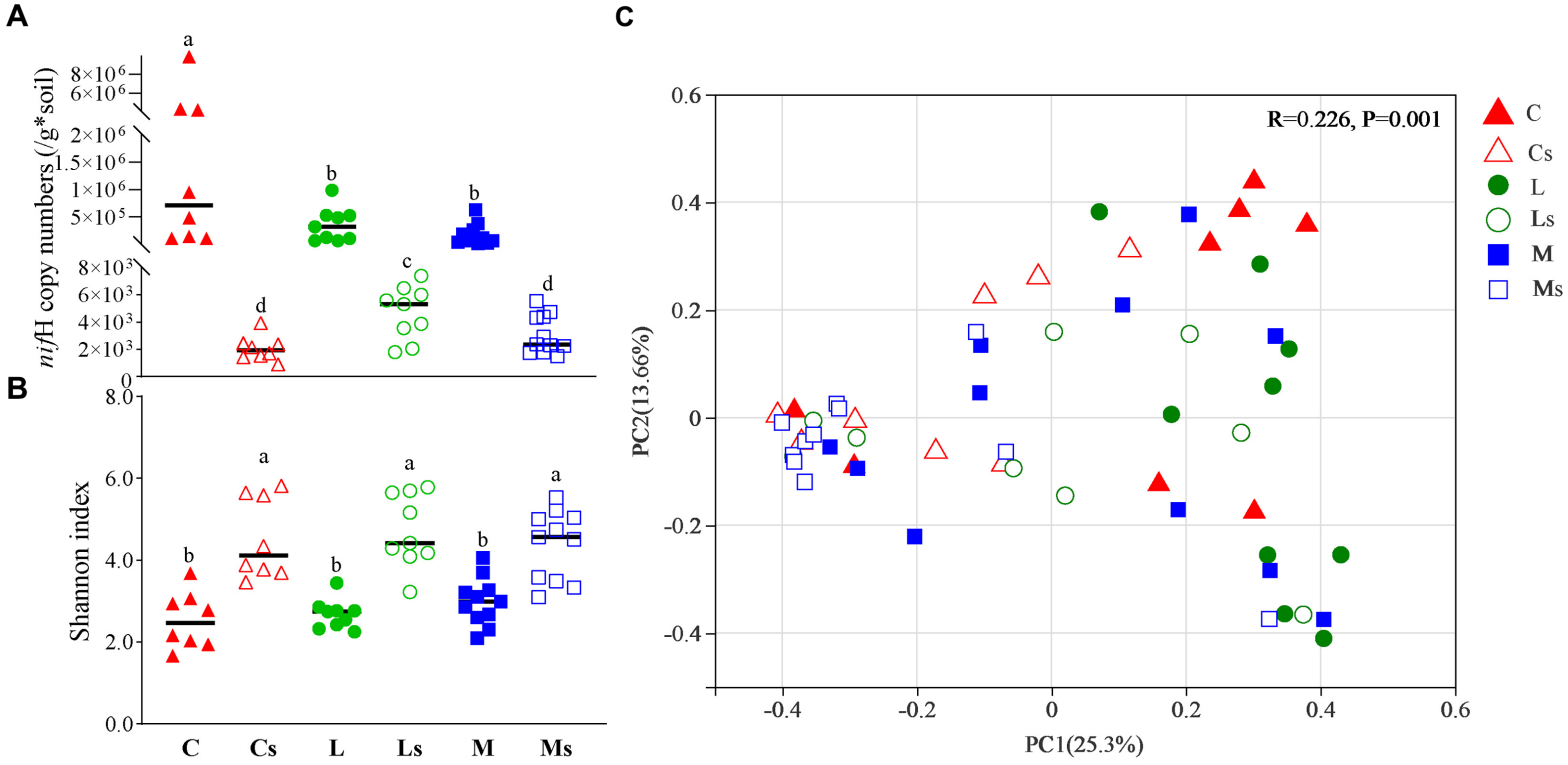
*nif*H copy number **(A)**, Shannon index **(B)**, and community structure **(C)** for diazotroph communities. Triangles, circles, and squares represent Cyano-, Lichen-, and Moss-BSCs communities, respectively. The points in rectangular represented are the samples collected from Badain Jaran desert. The *nif*H copy number differences between groups were analyzed by one-way ANOVA. The PCoA was based on Bray-Curtis distances, and group differences were evaluated with an ANOSIM test (*n* = 999). C, L, and M correspond to Cyano-, Lichen-, and Moss-BSCs, respectively. An “s” letter in the sample name indicates subsoil samples.

The *nif*H copy numbers were significantly different (*p* < 0.05) in BSCs and their subsoils, varying from 1.23 × 10^4^ to 9.88 × 10^6^ in BSCs and from 8.94 × 10^2^ to 7.41 × 10^3^ in subsoils. The average copy number of *nif*H in BSCs was above 10^5^, about two orders of magnitude greater than that in subsoils with significant difference (*p* < 0.05). Higher numbers were observed in Cyano-BSCs than in the other two successional stage BSCs, but slightly greater numbers appeared in subsoils of Lichen-BSCs at similar locations (*p* < 0.05) (Figure 3A).

### 3.3. Diversity of diazotrophs in BSCs and subsoils

A total of 1,615,168 *nif*H gene sequences were retrieved with average lengths of 427 bp, and 31,593 OTUs were generated using a threshold of 95% similarity. The Shannon indexes of subsoils were significantly higher than those of BSCs (*p* < 0.05) (Figure 3B). Except for Cyano-BSCs and Moss-BSCs samples collected from the Mu Us desert (Figure 1 and S5 site), most samples showed similar variation trends of Shannon indexes, i.e., being higher in BSCs, the index was simultaneously higher in the corresponding subsoils and vice versa. For the location nearest- distance sample, the indexes of both BSCs and subsoils became greater followed the succession order from Cyano-, Lichen- to Moss-BSCs. The Shannon index among different types of BSCs had no significant difference (*p* < 0.05) but increased slightly from Cyano- to Lichen- and Moss-BSCs. The similar shifts of Shannon index in subsoils were observed (Figure 3B).

### 3.4. Community structure of diazotrophs in BSCs and subsoils

The diazotroph bacterial community structures in the three types of BSCs and their subsoils were different according to principal coordinates analysis (PCoA) and analysis of similarity (ANOSIM) based on OTUs based on the OTU level (*R* = 0.226, *p* = 0.001) (Figure 3C and Supplementary Figures S1A–C). From in the same sampling regions, there was similar community structures of diazotroph bacteria between Cyano- and Lichen-BSCs (Supplementary Figures S1A,B,D). Notably, diazotroph communities were very similar in the three types of BSCs and their corresponding subsoils in the Badain Jaran desert (Figure 1 and S1 site), where the mean annual precipitation was lower than 100 mm (Figure 3C). In addition, the diazotroph community had no significant difference among subsoils beneath different BSCs (Supplementary Figure S1E).

The taxa numbers in subsoils beneath different BSCs were varied at the taxonomic levels of phylum, class, order, family, genus, and species. There were the largest numbers at each taxon level (Phylum: 7, Class: 13, Order: 21, Family: 31, Genera: 42, and Species: 52) in subsoil of Moss-BSCs (Supplementary Table S2). Besides the shared two known phyla (Cyanobacteria and Proteobacteria), three known classes (Alpha-, Beta-, and Gamma-proteobacteria), and eight known genera (*Nostoc*, *Azohydromonas*, *Azospirillum*, *Skermanella*, *Scytonema*, *Anabaena*, *Trichormus*, and *Rhodomicrobium*), the numbers of unique genera in subsoils also were much higher than that in BSCs (Supplementary Figure S2). The phyla of Cyanobacteria and Proteobacteria dominated in both BSCs and subsoils, with the most dominant ratios of Cyanobacteria in Cyano-BSCs and Lichen-BSCs (up to 87.50% in Lichen-BSCs) and of Proteobacteria in subsoils (up to 82.02% in the subsoil of Moss-BSCs). The genus *Scytonema* was dominant (53.21%) in Cyano-BSCs but decreased to 34.96% in Lichen-BSCs and to 16.29% in Moss-BSCs, while many unclassified bacterial genera appeared in higher BSCs succession stages. There were many more unclassified bacterial genera in subsoils, and the highest relative abundance was 26.17% in subsoils of Lichen-BSCs. The three types of BSCs and their subsoils shared four genera, one unclassified genus in Proteobacteria and three genera in the top 10, consisting of *Skermanella*, *unclassified_f__Rhodospirillaceae*, and *Scytonema* (Supplementary Figure S3). Besides these shared genera, there were three other genera, *Nostoc* and two unclassified genera in the order Nostocales and family Nostocaceae in BSCs, and *Azohydromonas* and two unclassified bacterial genera in subsoils. For average relative abundance, had the highest was 53.21% of *Scytonema* in Cyano-BSCs and the genus’ lowest abundance occurred in the subsoil of Moss-BSCs (2.31%). For individual samples, trace abundance was detected in samples collected from Badain Jaran and Hopq deserts (Figure 1 and S1 and S4 site). Nearly almost equal abundance in Moss-BSCs (36.76%) and their subsoils (36.51%) was *Skermanella*. In together, except for the unclassified groups, the genera *Skermanella*, *Scytonema*, *Azohydromonas*, *Nostoc*, and *Trichormus* were dominant genus in BSCs and their subsoils.

### 3.5. Interactions in diazotroph communities differed significantly but core microbiome was conserved among BSCs and their subsoils

Using random matrix theory (RMT)-based modeling with different threshold values (Supplementary Table S3), co-occurrence networks were constructed to discover the interaction in diazotroph communities. The obtained network sizes varied, but their links increased as well as the average degree (avgK) of each node (Cyano-BSCs: 20.11; Lichen-BSCs: 28.37; Moss-BSCs: 32.15) but average path distance (GD) decreased, following the BSCs successional stages ordered from Cyano-, Lichen- to Moss-BSCs. These suggested that the interactions in diazotroph communities were strengthened with more connections and shorter paths with other nodes in the network within higher BSCs stages. But for the interactions in the subsoils, bacterial networks in the subsoils of Lichen-BSCs had the highest numbers of links and avgK but had the lowest GD and average clustering coefficient (avgCC) and these in subsoils of Cyano- and Moss-BSCs were similar. These results indicated that the interactions in diazotroph communities differed significantly among BSCs and their subsoils.

**Figure 4:**
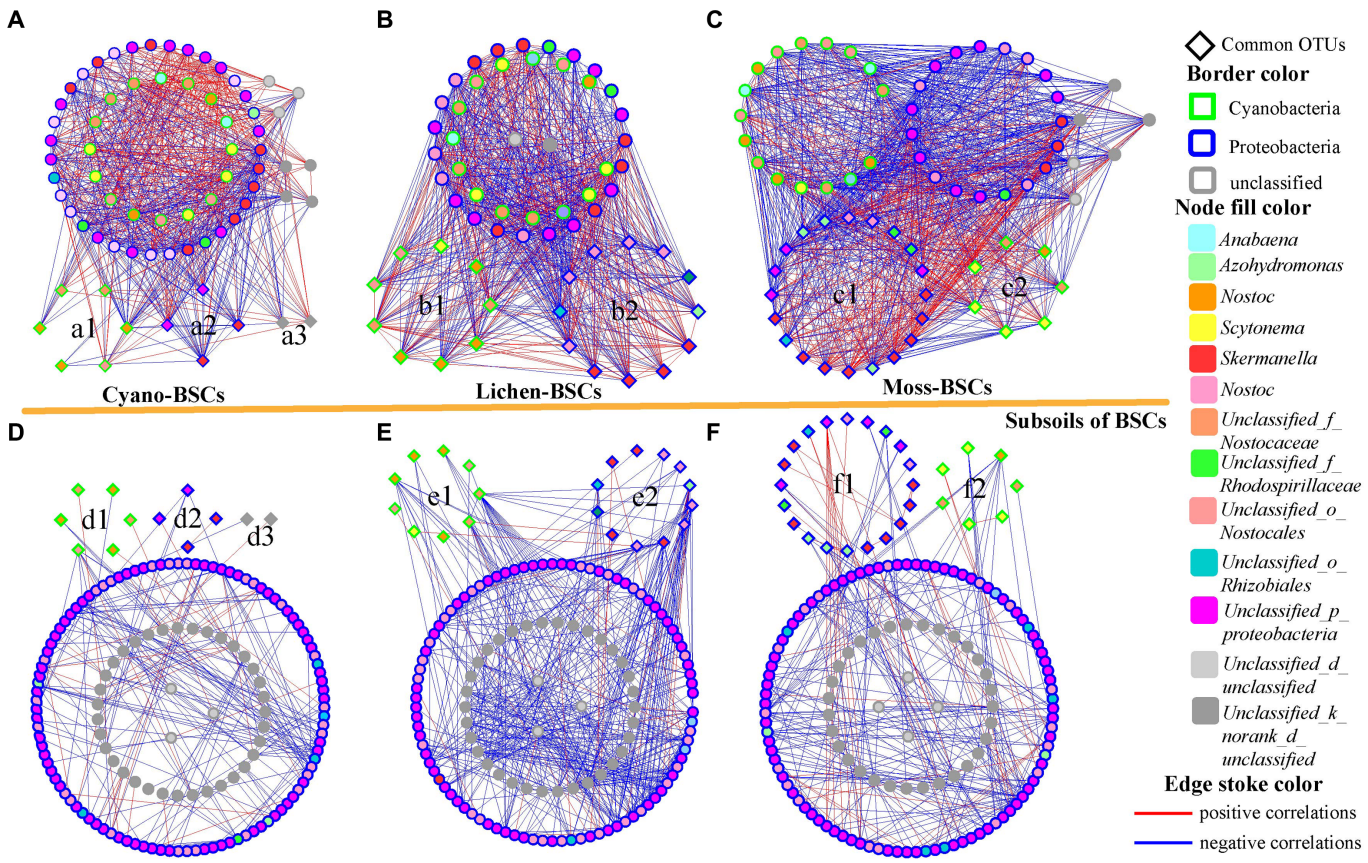
Network interactions of the diazotroph community based on *nif*H sequence OTU data. The networks were constructed with the RMT-based approach using the high-throughput data from Cyano- **(A)** and subsoil of Cyano-BSCs **(D)** (eight samples each), Lichen- **(B)** and subsoil of Lichen-BSCs **(E)** (nine samples each), and Moss- **(C)** and subsoil of Moss-BSCs **(F)** (eleven samples each). Nodes indicate different OTUs at 95% similarity. Colors of the nodes and borders indicate different genera and phyla. A blue line indicates a negative interaction between two individual nodes, while a red line indicates a positive interaction between two individual nodes.

The majority of nodes in the six networks belonged to two phyla and unclassified bacteria, but their distribution varied substantially among different phylogenetic groups (Figure 4 and Supplementary Table S4). Proteobacteria and Cyanobacteria were dominant phyla in all groups; the number of nodes belonging to Proteobacteria increased with the successional development of BSCs (Cyano-BSCs: 31.43%; Lichen-BSCs: 58.33%; Moss-BSCs: 59.09%), and Cyanobacteria decreased to 34.85% in Moss-BSCs. In subsoils, the percentage of nodes belonging to Proteobacteria was about 70%, and the percentage of nodes belonging to Cyanobacteria was about 22–24% (Supplementary Table S4). Except for the unclassified groups, *Skermanella*, *Nostoc*, and *Scytonema* were the three dominant genera in BSCs; their relative abundances were 16.42–18.64%, 7.46–8.47%, and 7.46–8.47%, respectively, and these were stable following the development of BSCs. In subsoils, the genus of *Skermanella* had the highest abundance in Moss-BSCs, being 2.56 and 3.93 times that in Lichen- and Cyano-BSCs, respectively. The relative abundance of *Scytonema* also increased BSCs in subsoils of higher BSCs successional stages (subsoils of Cyano-BSCs: 0.00%; subsoils of Lichen-BSCs: 0.68%; subsoils of Moss-BSCs: 2.61%).

Cyanobacteria and Proteobacteria were the common phyla in different types of BSCs and their corresponding subsoils, especially the genera *Scytonema*, *Nostoc* and *Skermanella*. In Cyano-BSCs and their subsoils, six OTUs belonged to Cyanobacteria (Figures 4a1,d1); four OTUs belonged to Proteobacteria (Figures 4a2,d2), and the other two unclassified OTUs (Figures 4a3,d3) were common. Among the three OTUs were members of the genus *Nostoc*, and two OTUs belonged to the genus *Skermanella*. The percentages of common OTUs in Lichen-BSCs and their subsoils belonging to *Nostoc* (Figures 4b1,e1) and *Skermanella* (Figures 4b2,e2) were the same. Except for several unclassified OTUs, three common OTUs belonged to the genera *Scytonema*, *Azohydromonas*, and *Pseudomonas*. In Moss-BSCs and their subsoils, twenty common OTUs belonged to Proteobacteria; eight of these were members of the genus *Skermanella* (Figures 4c1,f1). There were seven common OTUs belonging to Cyanobacteria; four belonged to *Scytonema*, and only one OTU belonged to the genus *Nostoc* (Figures 4c2,f2). Thus, *Scytonema*, *Nostoc* and *Skermanella* were common and dominant genera in all BSCs developmental stages and their corresponding subsoils, and were defined as the core microbiome in diazotroph communities of BSCs and their subsoils.

### 3.6. Core microbiome of diazotroph communities in BSCs and their subsoils had close relationships with available nitrogen content

Structural equation model was applied to disclose the relationships of core microbiome with AN. The fitted models explained 85% of the variance in diazotroph community composition in BSCs and their subsoils (Supplementary Figure S4), and diazotrophs belonging to Nostocaceae and Scytonemataceae had the highest explanatory power (*R*^2^ = 0.19, *R*^2^ = 0.51) as members of the core diazotroph community. Core diazotrophs (belonging to Nostocaceae and Scytonemataceae) were directly affected by the AN (*r* = 0.35, *p* = 0.005; *r* = 0.22, *p* = 0.026), SOC (*r* = 0.25, *p* = 0.042; *r* = 0.20, *p* = 0.042), and the diazotroph population [the relative abundance of Scytonemataceae was also influenced by the copy number of *nif*H (*r* = 0.65, *p* < 0.001)], which in turn showed significant correlations with their community composition. Standardized total effects derived from the SEM indicated that core diazotrophs (belonging to Nostocaceae and Scytonemataceae) could determine the diazotroph communities, also had tightly correlations with the AN content.

MaAsLin analysis identified core diazotroph communities (belonged to *Scytonema* and *Skermanella*) were significantly associated with the AN content in three types of BSCs and subsoils (Table 1). The dominant species of *Nostoc commune* and unclassified_g__*Nostoc* in *Nostoc* had significant correlation with the AN content, although diazotrophs belonged to the *Nostoc* had not in genus level. Diazotrophs belong to *Skermanella* were significantly negative with the accumulation of AN content in genus level, but two *Skermanella* sp. operational taxonomic units (OTUs) had positive correlation with it (Table 1).

**Table 1:** Core diazotrophs were significantly associated with the available nitrogen content of three types of Biological soil crusts (BSCs) and their subsoils in different taxonomy level.

Taxonomy		Coefficient (x 10^−3^)	*P*-value	*Q*-value
Genus	*Scytonema*	8.62	0.043	0.064
	*Nostoc*	1.96	0.255	0.255
	*Skermanella*	−9.41	0.035	0.064
Species	*Nostoc commune*	1.72	0.016	0.036
	*Nostoc punctiforme*	0.73	0.260	0.304
	Unclassified_g__*Nostoc*	1.35	0.014	0.036
	*Scytonema* sp. NC-4B	2.44	0.348	0.348
	*Scytonema* sp. NCC-4B	4.34	0.035	0.050
	Unclassified_g__*Scytonema*	5.73	0.004	0.025
	*Skermanella aerolata*	−9.41	0.035	0.050
OTU	*Nosto*c sp. OTU24229	1.73	0.016	0.081
	*Nostoc* sp. OTU24305	1.01	0.007	0.054
	*Nostoc* sp. OTU19070	0.40	0.010	0.069
	*Nostoc* sp. OTU6898	0.38	0.038	0.143
	*Scytonema* sp. OTU4853	4.52	0.003	0.031
	*Scytonema* sp. OTU23847	4.35	0.035	0.141
	*Scytonema* sp. OTU8608	2.75	0.002	0.023
	*Scytonema* sp. OTU2340	2.36	0.002	0.023
	*Scytonema* sp. OTU265	1.27	0.001	0.016
	*Skermanella* sp. OTU19491	1.40	0.002	0.023
	*Skermanella* sp. OTU25841	1.16	0.014	0.079
	*Skermanella* sp. OTU13772	−1.10	0.002	0.023
	*Skermanella* sp. OTU6896	−0.46	0.038	0.143
	*Skermanella* sp. OTU9221	−0.50	0.004	0.033

## 4. Discussion

BSCs are the living skin of the desert, and as such, they play a vital role in driving key biogeochemical cycles. They are the primary source of nitrogen (N) through N-fixation (Elbert et al., 2012; Zhou et al., 2020; Ramond et al., 2022), which could alleviate the lacking of soil N in desert ecosystems. The diazotroph communities in BSCs are critical groups, as they control the nitrogen fixation ability of BSCs and their subsoils.

### 4.1. Diazotroph communities of BSCs had lower absolute abundance, higher diversity, and more varied relative abundance than those of their subsoils

The *nif*H copy number of diazotrophs in BSCs was almost 100 times higher than that in their corresponding subsoils, a pattern that may be due to the contents of soil nutrient substances (soil carbon, available phosphorus and available nitrogen content) being higher in BSCs (Tang et al., 2021). Low abundance of the genes involved in N_2_ fixation pathways is a distinguishing feature of BSCs microbiomes (Li et al., 2020), but diazotroph communities had higher Shannon diversity in the subsoils of BSCs in our results. This may have been due to desert soil carbon and nitrogen availability is generally low (Delgado-Baquerizo et al., 2013), and non-motile N_2_-fixing heterocystous cyanobacteria occupying the BSCs surface, which led to other microbial groups be reduced. They could accumulate soil carbon and nitrogen (Pepe-Ranney et al., 2015), that could support more heterotrophic microbes alive. Moreover, heterocystous cyanobacteria could produce large amounts of sunscreening compounds that reduced the soil albedo (Belnap, 2002; Yeager et al., 2004), which also maybe the reason why the subsoil had higher diversity than their corresponding BSCs. The abundance of diazotrophs was higher in western with lower mean annual precipitation, then leaded to AN content was higher in the Badain Jaran desert than in the Mu Us desert, that’s may due to soil type of sampling sites (Zhou et al., 2021).

All known diazotrophs were belonged to bacteria and Archaea (Kuypers et al., 2018). In our results, diazotroph communities were diverse, largely distributed in the phyla Proteobacteria and Cyanobacteria with more than 50 species. The dominant phyla were Proteobacteria and Cyanobacteria, and these also had the most common genera (*Skermanella*, *Scytonema*, *Nostoc*, and *Azohydromonas*) in relative abundance. Proteobacteria (56%) and Cyanobacteria (10%) were the most abundant soil nitrogen-fixers (Gaby and Buckley, 2011). *Unclassified bacteria* were largely N-fixing groups in the Tengger desert (Hu et al., 2019), and the genus *Azotobacter* comprised diazotrophs in Hopq desert (Zhang et al., 2014). This differed from our results where *Skermanella*, *Scytonema*, *Nostoc*, *Azohydromonas*, and other unclassified bacterial genera were the main genera of all subsoils in different BSC developmental stages in the Inner Mongolian plateau. However, most studies have indicated that *Nostoc* spp., *Scytonema* spp., and *Tolypothrix/Spirirestis* spp. are the main genera in BSCs of the Colorado plateau and the Great Basin desert (Yeager et al., 2004, 2007; Zhou et al., 2016), in agreement with our results. Cyanobacteria are autotrophic, and nitrogen-fixing Nostocales can survive in many extreme situations (Dodds et al., 1995) and dominate the diazotroph communities in the barren soils of arid and semi-arid climates (Bhatnagar et al., 2008; Yeager et al., 2012; Dojani et al., 2014). This may explain why highly abundant nitrogen-fixing Nostocales were identified in desert BSCs. In addition, the diazotroph community in different types of BSCs share similar kinds of genus, only differ in abundance (Liu et al., 2019). This pattern was also shown in subsoils in our results.

In summary, the diazotroph communities in different types of BSCs had higher absolute abundance and lower diversity than their corresponding subsoils. Proteobacteria and Cyanobacteria were the dominant phyla; *Skermanella*, *Scytonema*, and *Nostoc* (except for the unclassified groups) were the dominant genera and were distributed in all samples, and the main difference was in the relative abundance.

### 4.2. Available nitrogen content was related to the core diazotroph communities in the three types of BSCs and their subsoils

The *nif*H absolute abundance showed significant differences among the types of BSCs and their subsoils, but this did not match the pattern of AN content, possibly due to some bacteria being unable to fix nitrogen, even though they had nitrogen-fixing genes in their genomes (Subhash and Lee, 2016; Subhash et al., 2017). As these organisms have different N fixation rates, it is expected that the N fixation capability of the BSCs community will change with variation in species composition. Wang et al. (2016) revealed Proteobacteria-like diazotrophs predominated in the mobile dunes, while the cyanobacterial diazotrophs predominated in the revegetated sites. As BSCs and subsoils owing the highest AN content was not congruent, and that of Moss-S (Ms) was generally higher than that of Cyano-S (Cs) and Lichen-S (Ls), AN of subsoils maybe be partially and importantly derived from nitrogen fixation by its own diazotrophs and, non-photoauototrophic, non-cyanobacterial and carbon-heterotrophs turned to be prominent diazotrophs. Thus, core diazotrophs should be exist in all diazotroph communities, and they could fix nitrogen and increase AN content of BSCs and their subsoils.

Core microbiome were considered as an active and instrumental part of the ecosystem and function of the host or environment (Banerjee et al., 2018; Yang et al., 2020). There is currently no uniform method for defining the core microbiome (Risely, 2020), but their members should be shared taxa, having certain function and hub nodes in communities. Microbial cooccurrence network was employed to predict the core diazotrophs in BSCs and subsoil, and found OTUs belonged to the genera *Nostoc*, *Scytonema* and *Skermanella* were identified as the hub nodes in BSCs and their subsoils’ diazotroph community network, except to unclassified groups. Meanwhile, they were involved in nitrogen fixation, and showed significant correlations with the AN content in the development of BSCs (Table 1), so species belonged to the three genera were considered as the core diazotrophs. Among, heterocystous cyanobacteria (*Scytonema* and *Nostoc*) are widely recognized as important N_2_-fixing components of BSCs (Yeager et al., 2007, 2012; Pepe-Ranney et al., 2015). *Scytonema* spp. tend to be more thermotolerant (Giraldo-Silva et al., 2020), that’s why they had high abundance and could be suitable for the Inner Mongolian plateau compared to the other Cyanobacteria nitrogen-fixing groups. They were the most common heterocystous organisms and were proved could contribute much of the nitrogen input to different types of BSCs (Giraldo-Silva et al., 2020). *Nostoc* spp. showed psychrophilic preference for mild temperatures, so they had lower abundance in BSCs and subsoils. *Skermanella* spp. are purple non-sulfur bacteria that are capable of photosynthesizing, and they can switch among photoheterotrophic, photoautotrophic, and chemoheterotrophic strategies depending on soil aeration, soil carbon, and light availability (Imhoff et al., 2019), so they had stable abundance in BSCs, even increased in subsoils. And that’s why they could survive in Cyano-BSCs with low organic matter content. Nitrogen fixation was a high energy-consuming process where each molecule of nitrogen fixed consumes 16 molecules of ATP (Bothe et al., 2010). Species in the genera *Scytonema* and *Nostoc* are photoautotrophic; species in *Skermanella* are mixotrophic, so species in three genera had advantage in fixing N_2_ in low organic matter desert than the heterotrophic groups. That also maybe a reason of the three genera could be the core diazotrophs.

Thus, species belonged to the genera *Nostoc*, *Scytonema* and *Skermanella*, had tightly correlation with the AN content, they were the core diazotrophs in the succession of BSCs and their subsoils.

## 5. Conclusion

This study demonstrated that the diazotroph communities have higher abundance in BSCs and higher diversity in their subsoils. Proteobacteria and Cyanobacteria were the dominant phyla, and the genera *Skermanella, Scytonema, Azohydromonas, Nostoc*, *and Trichormus* were the main genera of diazotrophs. Diazotroph communities had different interactions in three types of BSCs and their subsoils, and species belonging to the genera of *Skermanella*, *Scytonema*, and *Nostoc* were significantly correlated with AN content. Collectively, the diazotroph community in BSCs and their subsoils had high diversity; *Skermanella*, *Scytonema*, and *Nostoc* were the core microbiome of diazotrophs, they were also the main contributor to nitrogen fixation in BSCs and subsoils in temperate deserts of Inner Mongolia plateau.

## Data availability statement

The datasets presented in this study can be found in online repositories. The names of the repository/repositories and accession number(s) can be found in the article/Supplementary material.

## Author contributions

KT carried out the experiments and prepared the manuscript draft. KT, YL, and BY analyzed the data. YL and BY helped in the sampling from deserts and preparing the experiments. JM and FF designed the research and wrote the manuscript with input from KT. All authors contributed to the article and approved the submitted version.

## Funding

This study was financially supported by the Program of the National Natural Science Foundation of China (32001220), the Applied Technology Research and Development Fund of Inner Mongolia (2021GG0360), and Inner Mongolia Agricultural University High-level/Outstanding Doctoral Introduction Talents Research Start-up Project (NDYB 2021-24), Program for improving the Scientific Research Ability of Youth Teachers of Inner Mongolia Agricultural University (BR230149).

## Conflict of interest

The authors declare that the research was conducted in the absence of any commercial or financial relationships that could be construed as a potential conflict of interest.

## Publisher’s note

All claims expressed in this article are solely those of the authors and do not necessarily represent those of their affiliated organizations, or those of the publisher, the editors and the reviewers. Any product that may be evaluated in this article, or claim that may be made by its manufacturer, is not guaranteed or endorsed by the publisher.

## Footnotes

^1^ http://data.cma.cn/site/index.html

^2^ http://drive5.com/uparse/

^3^ http://rdp.cme.msu.edu/
