# Supplementary material for: Spatial distribution and core community of diazotrophs in Biological soil crusts and subsoils in temperate semi-arid and arid deserts of China
Source: Front Microbiol. 2023 Aug 7;14:1074855. doi: 10.3389/fmicb.2023.1074855 (PMC10440438; doi:10.3389/fmicb.2023.1074855)
Supplement: Supplementary file 1 [file Data_Sheet_1.docx]

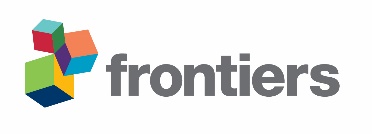


Supplementary Material

**Spatial distribution and core community of diazotrophs in Biological soil crusts and subsoils in temperate semi-arid and arid deserts of China**

Kai Tang^1^, Yungang Liang^1^, Bo Yuan^2, 1^, Jianyu Meng^1*^, Fuying Feng^1*^

^1^ Laboratory for Environmental Microbiology and Biotechnology in Arid and Cold Regions, College of Life Sciences, Inner Mongolia Agricultural University, Hohhot 010018, PR China.

^2^ College of Life Science, Inner Mongolia Normal University, Hohhot 010018, PR China.

*Correspondence: Jianyu Meng, meng_jianyu@imau.edu.cn; Fuying Feng, foyefeng@hotmail.com

**Supplementary Tables**

**Table S1.** The collection months, geographic locations, and the AN content for 56 samples of BSCs and their subsoils. The AN content was shown as mean ± SD of three independent experiments. In sample names, “s” and “m” in small case in the front indicating the sampling time of September and May; A, L and M in the rear in capital case indicating sampled from Cyano-, Lichen- and Moss-BSCs and their corresponding subsoils, respectively. In Tengger desert, two subsites distanced from about 5.0 kilometer were labelled with “1” and “2” in the sample names.

**Table S2.** Overview of diazotroph communities in biological soil crusts and their subsoils.

**Table S3.** Key parameters of network topology about network modules and interactions of nitrogen-fixing bacteria based on *nif*H sequence OTUs data. An ‘S’ in the sample name indicates subsoil samples.

**Table S4.** Summary of the network complexity of various phylogenetic groups. The number indicates the number of OTUs in network. An ‘S’ in the sample name indicates subsoil samples.

**Supplementary Figures**

**Figure S1** Community structure in C and Cs (a), L and Ls (b), M and Ms (c), C/L/M (d), and Cs/Ls/Ms (e) for diazotroph communities. Triangles, circles, and squares represent Cyano-, Lichen-, and Moss-BSC communities, respectively. The PCoA was based on Bray-Curtis distances, and group differences were evaluated with an ANOSIM test (n = 999). C, L, and M correspond to Cyano-, Lichen-, and Moss-BSCs respectively. An “s” letter in the sample name indicates subsoil samples.

**Figure S2** Veen for three types of BSCs and their subsoils at the genus level. The number in middle circle shows consensus families from three types of BSCs and their subsoils. The other numbers denote unique families in different groups.

**Figure S3**. Community structure and composition of diazotroph bacteria in the biological soil crusts and their subsoils (at the level of genus). Groups of less than 0.1% were classified as others. C, L and M correspond to Cyano-, Lichen- and Moss-BSCs respectively. An ‘s’ letter in the sample name indicates subsoil samples.

**Figure S4** Structural equation models showing the direct effects of available nitrogen (AN), soil organic carbon (SOC) content, *nif*H copy numbers, relative abundance of Nostocaceae and Scytonemataceae on diazotroph community composition. Numbers adjacent to arrows are path coefficients, continuous arrows indicate positive relationships.

**Table S1.** The collection months, geographic locations, and the AN content for 56 samples of BSCs and their subsoils. The AN content was shown as mean ± SD of three independent experiments. In sample names, “s” and “m” in small case in the front indicating the sampling time of September and May; A, L and M in the rear in capital case indicating sampled from Cyano-, Lichen- and Moss-BSCs and their corresponding subsoils, respectively. In Tengger desert, two subsites distanced from about 5.0 kilometer were labelled with “1” and “2” in the sample names.

| **Sites** | **Samples** | **Geographic location** | **AN (mg×kg^-1^)** | | **Sites** | **Samples** | **Geographic location** | **AN (mg×kg^-1^)** | |
| --- | --- | --- | --- | --- | --- | --- | --- | --- | --- |
|  |  |  | **BSCs** | **subsoil** |  |  |  | **BSCs** | **subsoil** |
| Badain Jaran desert (S1) | mBDL | 39.426°N, 100.977°E | 52.57 ± 3.50 | 37.91 ± 1.39 | Hopq desert (S4) | mKA | 39.373°N, 109.501°E | 55.41 ± 0.18 | 29.57 ± 1.41 |
|  | sBDL | 39.426°N, 100.977°E | 73.5 ± 2.67 | 50.17 ± 2.77 |  | mKL | 39.373°N, 109.501°E | 43.74 ± 0.09 | 30.32 ± 1.41 |
|  | sBDM | 39.428°N, 100.984°E | 44.49 ± 1.75 | 34.49 ± 5.14 |  | mKM | 39.373°N, 109.501°E | 44.41 ± 1.23 | 36.24 ± 1.68 |
| Tengger desert (S2) | mT1L | 38.675°N, 105.624°E | 44.85 ± 1.01 | 32.67 ± 1.12 |  | sKA | 40.308°N, 108.404°E | 51.33 ± 1.06 | 30.33 ± 1.15 |
|  | mT1M | 38.675°N, 105.624°E | 39.07 ± 2.67 | 35.66 ± 4.15 |  | sKL | 40.308°N, 108.404°E | 51.49 ± 1.41 | 26.24 ± 1.56 |
|  | mT2A | 38.623°N, 105.559°E | 47.83 ± 2.67 | 25.67 ± 2.03 |  | sKM | 40.308°N, 108.404°E | 56.00 ± 1.15 | 43.17 ± 0.71 |
|  | mT2L | 38.623°N, 105.559°E | 58.33 ± 0.71 | 38.67 ± 1.94 |  | mBA | 40.226°N, 107.148°E | 31.50 ± 2.21 | 22.17 ± 1.36 |
|  | mT2M | 38.623°N, 105.559°E | 48.41 ± 2.41 | 40.66 ± 1.41 |  | mBL | 40.226°N, 107.148°E | 51.03 ± 1.59 | 32.05 ± 1.01 |
|  | sT1L | 38.675°N, 105.624°E | 53.66 ± 3.12 | 25.24 ± 2.03 |  | mBM | 40.226°N, 107.148°E | 42 ± 1.15 | 38.5 ± 0.84 |
|  | sT1M | 38.6753°N, 105.624°E | 78.17 ± 0.34 | 43.17 ± 1.32 |  | sBA | 40.226°N, 107.148°E | 32.67 ± 1.32 | 26.83 ± 0.37 |
|  | sT2A | 38.623°N, 105.559°E | 34.33 ± 4.77 | 21.5 ± 1.85 |  | sBL | 40.226°N, 107.148°E | 35.00 ± 1.59 | 18.67 ± 0.24 |
|  | sT2M | 38.623°N, 105.559°E | 70.20 ± 4.37 | 38.09 ± 1.06 |  | sBM | 40.226°N, 107.148°E | 52.50 ± 1.32 | 51.33 ± 0.24 |
| Wulanbuhe desert (S3) | sCA | 39.894°N, 106.314°E | 33.24 ± 0.18 | 30.32 ± 1.41 | Mu Us desert (S5) | sMSA | 39.373°N, 109.501°E | 16.32 ± 1.76 | 13.41 ± 1.32 |
|  | sCM | 39.894°N, 106.314°E | 53.07 ± 0.05 | 37.49 ± 1.23 |  | sMSM | 39.373°N, 109.501°E | 53.10 ± 2.12 | 39.02 ± 1.85 |

**Table S2.** Overview of diazotroph communities in biological soil crusts and their subsoils.

| Groups | Phylum | Class | Order | Family | Genus | Species |
| --- | --- | --- | --- | --- | --- | --- |
| C | 4 | 9 | 12 | 19 | 26 | 31 |
| Cs | 6 | 10 | 18 | 27 | 37 | 45 |
| L | 3 | 7 | 11 | 16 | 22 | 27 |
| Ls | 6 | 10 | 14 | 23 | 31 | 40 |
| M | 4 | 9 | 17 | 27 | 37 | 42 |
| Ms | 7 | 13 | 21 | 31 | 42 | 52 |

Note: OTUs, operational taxonomic units; C, L and M correspond to Cyano-, Lichen-, and Moss-BSCs, respectively. An ‘s’ in the sample name indicates subsoil samples.

**Table S3.** Key parameters of network topology about network modules and interactions of nitrogen-fixing bacteria based on *nif*H sequence OTUs data. C, L, and M correspond to Cyano-, Lichen-, and Moss-BSCs, respectively. An ‘s’ in the sample name indicates subsoil samples.

| **Groups** | **RMT**  **threshold** | **Total**  **nodes** | **Total**  **Links** | **Average degree**  **(****avgK)** | **Average**  **path distance (GD)** | **Average clustering**  **coefficient (avgCC)** |
| --- | --- | --- | --- | --- | --- | --- |
| C | 0.610 | 70 | 704 | 20.114 | 1.775 | 0.467 |
| Cs | 0.980 | 153 | 236 | 3.085 | 4.406 | 0.128 |
| L | 0.490 | 60 | 851 | 28.367 | 1.538 | 0.592 |
| Ls | 0.930 | 148 | 484 | 6.541 | 3.806 | 0.077 |
| M | 0.340 | 68 | 1093 | 32.147 | 1.520 | 0.538 |
| Ms | 0.850 | 157 | 295 | 3.758 | 4.474 | 0.112 |

**Table S4.** Summary of the network complexity of various phylogenetic groups. The number indicates the number of OTUs in network. C, L and M correspond to Cyano-, Lichen-, and Moss-BSCs, respectively. An ‘s’ in the sample name indicates subsoil samples.

| **Taxonomy** | | **Types of BSCs and their subsoils** | | | | | |
| --- | --- | --- | --- | --- | --- | --- | --- |
|  |  | **C** | **L** | **M** | **Cs** | **Ls** | **Ms** |
| Phylum | Proteobacteria | 22 | 35 | 39 | 107 | 102 | 115 |
|  | Cyanobacteria | 39 | 23 | 23 | 6 | 9 | 7 |
|  | unclassified_k__norank_d__Bacteria | 9 | 2 | 4 | 37 | 35 | 35 |
| Family | Rhodospirillaceae | 13 | 13 | 14 | 3 | 5 | 10 |
|  | unclassified_p__Proteobacteria | 15 | 9 | 14 | 71 | 58 | 74 |
|  | unclassified_c__Alphaproteobacteria | 9 | 10 | 7 | 27 | 33 | 14 |
|  | unclassified_k__norank_d__Bacteria | 6 | 1 | 4 | 37 | 34 | 31 |
|  | Scytonemataceae | 5 | 5 | 5 | 0 | 1 | 4 |
|  | Alcaligenaceae | 1 | 1 | 3 | 2 | 1 | 5 |
|  | unclassified_o__Nostocales | 4 | 4 | 4 | 1 | 2 | 0 |
|  | Pseudomonadaceae | 0 | 1 | 0 | 1 | 1 | 0 |
|  | Nostocaceae | 13 | 13 | 14 | 5 | 6 | 3 |
|  | unclassified_o__Rhizobiales | 1 | 1 | 1 | 3 | 2 | 6 |
|  | Microchaetaceae | 0 | 1 | 0 | 0 | 0 | 0 |
|  | Hyphomicrobiaceae | 0 | 0 | 0 | 0 | 2 | 1 |
| Genus | *Anabaena* | 2 | 1 | 2 | 0 | 0 | 0 |
|  | *Skermanella* | 11 | 11 | 11 | 2 | 5 | 8 |
|  | unclassified_p__Proteobacteria | 15 | 9 | 14 | 71 | 58 | 74 |
|  | unclassified_c__Alphaproteobacteria | 9 | 10 | 7 | 27 | 33 | 19 |
|  | unclassified_k__norank_d__Bacteria | 6 | 1 | 4 | 37 | 34 | 31 |
|  | *Scytonema* | 5 | 5 | 5 | 0 | 1 | 4 |
|  | *unclassified_f__Rhodospirillaceae* | 2 | 2 | 3 | 1 | 0 | 2 |
|  | *Azohydromonas* | 1 | 1 | 3 | 2 | 1 | 5 |
|  | *unclassified_o__Nostocales* | 4 | 4 | 4 | 1 | 4 | 0 |
|  | *Nostoc* | 5 | 5 | 5 | 3 | 4 | 1 |
|  | *unclassified_f__Nostocaceae* | 6 | 6 | 6 | 2 | 2 | 2 |
|  | *unclassified_o__Rhizobiales* | 1 | 1 | 1 | 3 | 2 | 6 |
|  | *Pseudomonas* | 0 | 1 | 0 | 0 | 1 | 0 |
|  | *Trichormus* | 0 | 1 | 1 | 0 | 0 | 0 |
|  | *Rhodomicrobium* | 0 | 0 | 0 | 0 | 2 | 1 |
|  | *Azotobacter* | 0 | 0 | 0 | 1 | 0 | 0 |


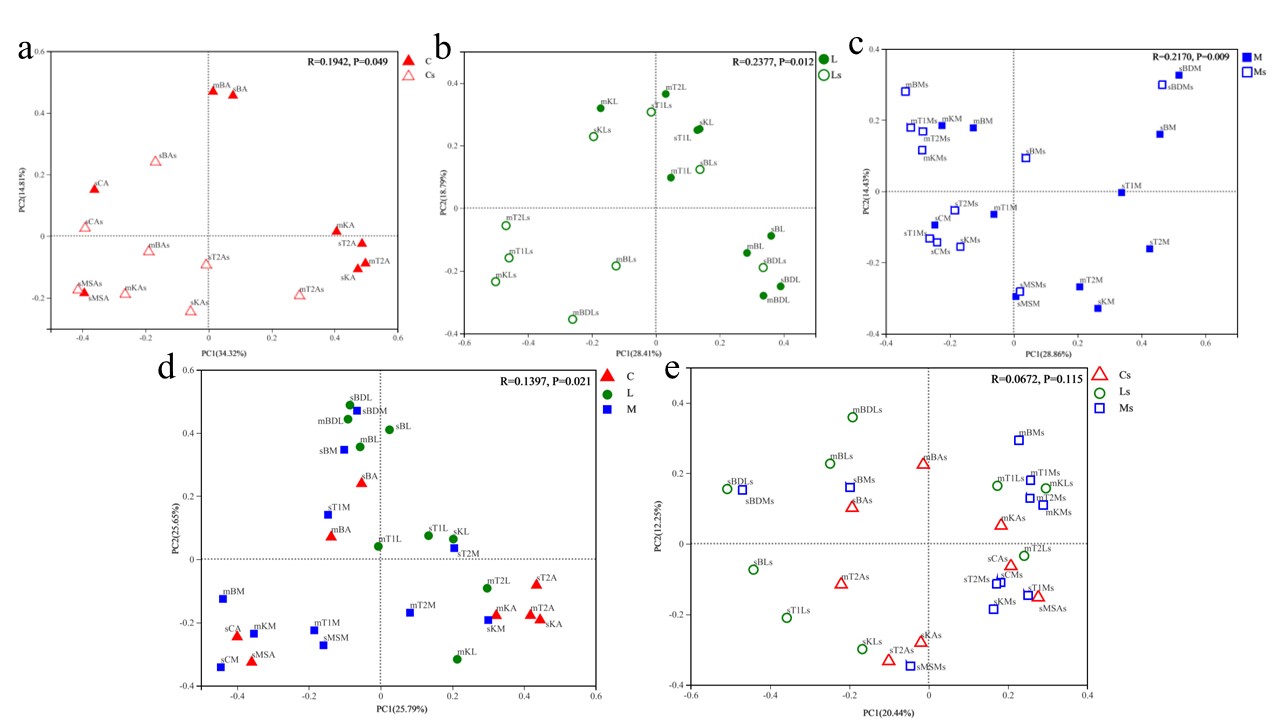


**Figure S1** Community structure in C and Cs (a), L and Ls (b), M and Ms (c), C/L/M (d), and Cs/Ls/Ms (e) for diazotroph communities. Triangles, circles, and squares represent Cyano-, Lichen-, and Moss-BSC communities, respectively. The PCoA was based on Bray-Curtis distances, and group differences were evaluated with an ANOSIM test (n = 999). C, L, and M correspond to Cyano-, Lichen-, and Moss-BSCs, respectively. An “s” letter in the sample name indicates subsoil samples.


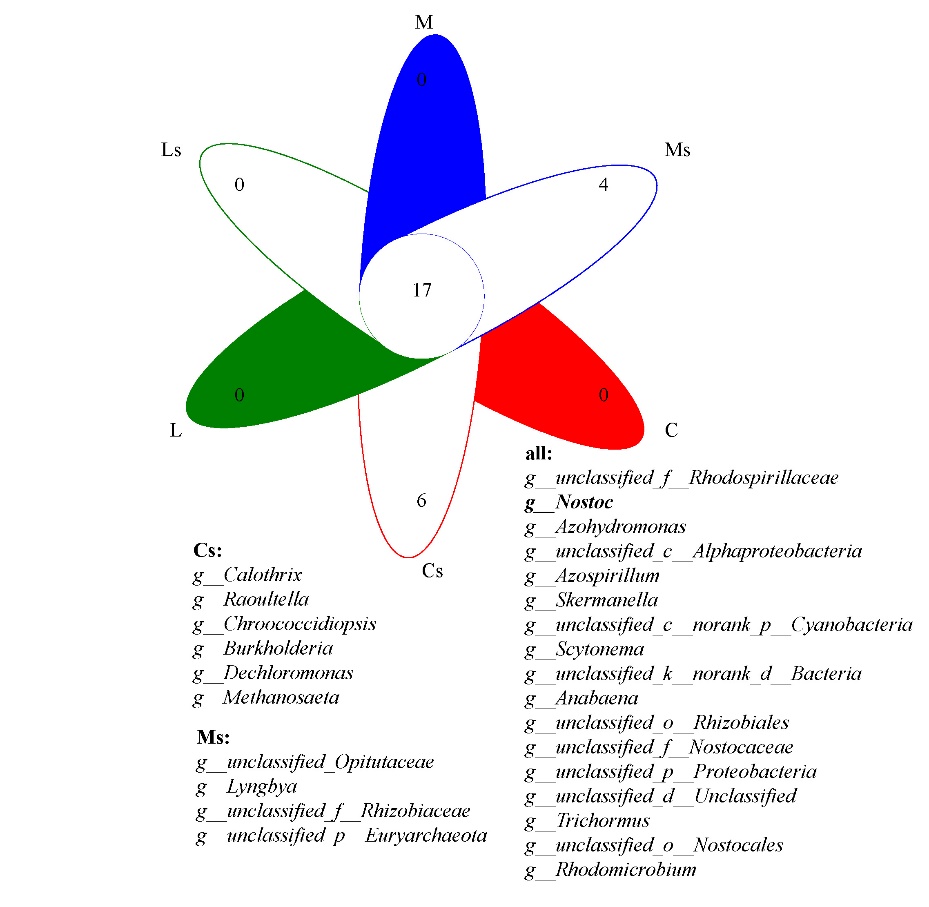


**Figure S2** Veen for three types of BSCs and their subsoils at the genus level. The number in middle circle shows consensus families from three types of BSCs and their subsoils. The other numbers denote unique genus in different groups.


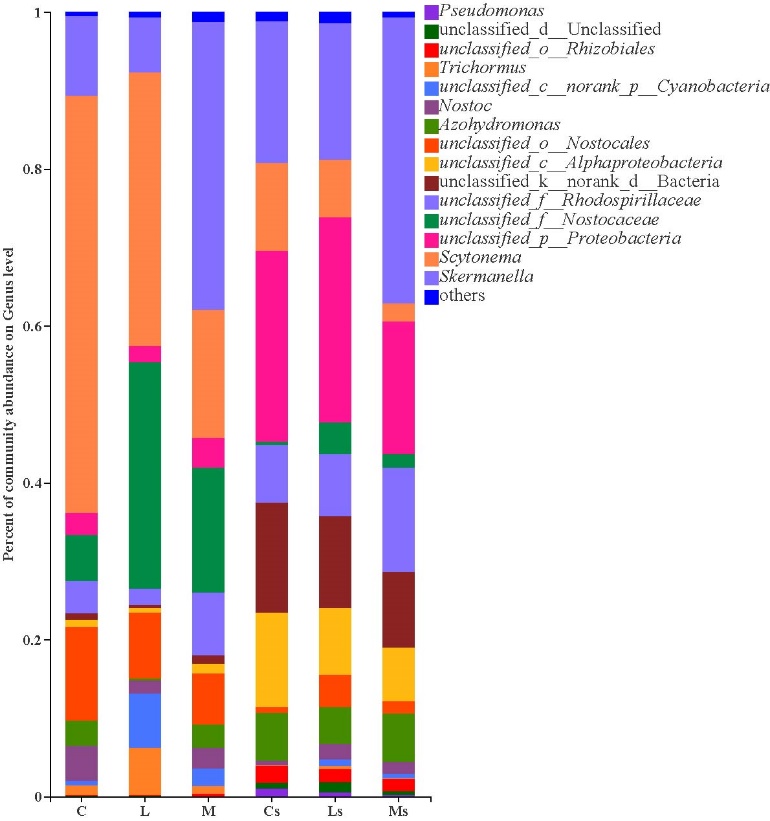


**Figure S3**. Community structure and composition of diazotroph bacteria in the biological soil crusts and their subsoils (at the level of genus). Groups of less than 0.1% were classified as others. C, L and M correspond to Cyano-, Lichen-, and Moss-BSCs, respectively. An ‘s’ letter in the sample name indicates subsoil samples.


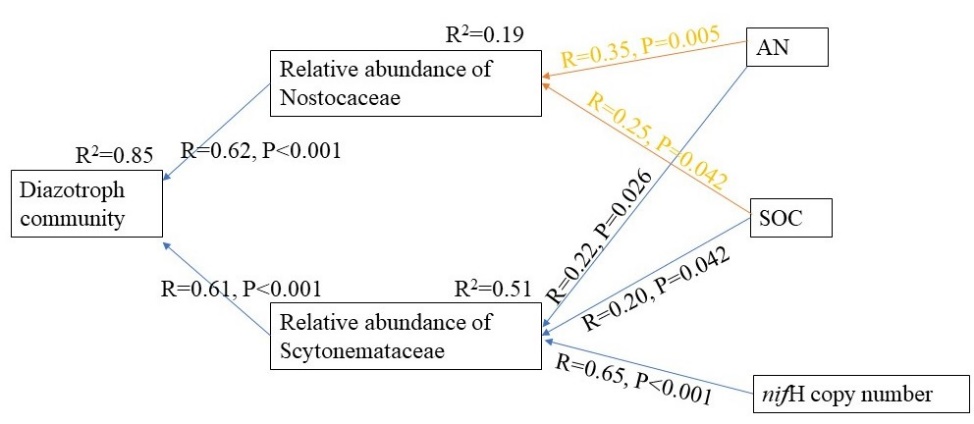


**Figure S4** Structural equation model showing the direct effects of available nitrogen (AN), soil organic carbon (SOC) content, *nif*H copy numbers, and relative abundances of Nostocaceae and Scytonemataceae on diazotroph community composition. Numbers adjacent to arrows are path coefficients, and continuous arrows indicate positive relationships.
